# 3D-manufactured patient-specific models of congenital heart defects for communication in clinical practice: feasibility and acceptability

**DOI:** 10.1136/bmjopen-2014-007165

**Published:** 2015-04-30

**Authors:** Giovanni Biglino, Claudio Capelli, Jo Wray, Silvia Schievano, Lindsay-Kay Leaver, Sachin Khambadkone, Alessandro Giardini, Graham Derrick, Alexander Jones, Andrew M Taylor

**Affiliations:** 1 Centre for Cardiovascular Imaging, Institute of Cardiovascular Science, University College London, London, UK; 2 Cardiorespiratory Division, Great Ormond Street Hospital for Children, NHS Foundation Trust, London, UK

## Abstract

**Objectives:**

To assess the communication potential of three-dimensional (3D) patient-specific models of congenital heart defects and their acceptability in clinical practice for cardiology consultations.

**Design:**

This was a questionnaire-based study in which participants were randomised into two groups: the ‘model group’ received a 3D model of the cardiac lesion(s) being discussed during their appointment, while the ‘control group’ had a routine visit.

**Setting:**

Outpatient clinic, cardiology follow-up visits.

**Participants:**

103 parents of children with congenital heart disease were recruited (parental age: 43±8 years; patient age: 12±6 years). In order to have a 3D model made, patients needed to have a recent cardiac MRI examination; this was the crucial inclusion criterion.

**Interventions:**

Questionnaires were administered to the participants before and after the visits and an additional questionnaire was administered to the attending cardiologist.

**Main outcome measures:**

Rating (1–10) for the liking of the 3D model, its usefulness and the clarity of the explanation received were recorded, as well as rating (1–10) of the parental understanding and their engagement according to the cardiologist. Furthermore, parental knowledge was assessed by asking them to mark diagrams, tick keywords and provide free text answers. The duration of consultations was recorded and parent feedback collected.

**Results:**

Parents and cardiologists both found the models to be very useful and helpful in engaging the parents in discussing congenital heart defects. Parental knowledge was not associated with their level of education (p=0.2) and did not improve following their visit. Consultations involving 3D models lasted on average 5 min longer (p=0.02).

**Conclusions:**

Patient-specific models can enhance engagement with parents and improve communication between cardiologists and parents, potentially impacting on parent and patient psychological adjustment following treatment. However, in the short-term, parental understanding of their child's condition did not improve.

**Strengths and limitations of this study**

- This study systematically quantifies usefulness and applicability of three-dimensional models produced with rapid prototyping technique.
- Models are typically used in case studies, whereas here a larger group of participants (>100) has been studied.
- A wide range of models of congenital heart disease was manufactured.
- The study shows liking from both users (ie, parents of patients and cardiologists), demonstrating feasibility of such communication method.
- The study raises potentially controversial points about current communication methods (eg, use of medical images difficult to interpret for the lay person) through feedback provided by participants.
- The study, however, targets only the parents; patients will be assessed in a separate study in the future.
- The study lacks a detailed cost analysis with regard to the applicability of the technique.

## Introduction

Three-dimensional (3D) engineered replicas of different anatomical structures have been used extensively in different fields of medicine over the past 20 years, including in orthopaedics,^1^ ^2^ maxillofacial surgery,^3–5^ cardiology^6^ and forensic medicine.^7^ As the manufacturing techniques—generally referred to as ‘rapid prototyping’—have become more refined over the years, medical researchers have used such 3D models for presurgical planning,^8^ personalisation of prostheses,^9^ ^10^ or testing of novel devices.^11^ Among the advocated benefits of anatomical 3D models, it has been suggested^12–14^ that being able to visualise the location and dimensions of the area of interest can aid in communication, both within a surgical team and, crucially, between the physician and the patient.

Communication is vitally important to doctors and patients,^15^ with aspects concerning the clarity of language, integrating frameworks, and researching the relationship between communication and interpersonal skills impacting on patient care.^16^ The complex nature of congenital heart disease (CHD) and the communication challenges it poses are particularly good examples of this. There is there an ‘expert to non-expert’ interaction, with little likelihood of shared knowledge between participants, as well as a situation where the unshared knowledge is highly technical and difficult to communicate verbally or with traditional media. It is well known that such circumstances increase the chances of unsuccessful communication.^17^

From a technical point of view, congenital heart defects frequently involve complex surgical operations and anatomical arrangements. Furthermore, for parents (ie, the non-expert individual) there is an additional emotional component of distress and anxiety further impinging on the exchange of knowledge during a consultation. 3D patient-specific models offer a potential new medium for improving communication in this challenging setting, improving parental understanding and possibly reducing the stress of the consultation.

The aim of this study was to produce a range of models of congenital heart lesions and test their impact on communication between parents and cardiologists. This study presents considerations about the feasibility and acceptability of manufacturing patient-specific 3D models for cardiac consultations, and their use in improving engagement of parents of children with CHD.

## Materials and methods

### Participants

One hundred and three parents of children with CHD who were attending clinics for routine outpatient follow-up were approached for the study. Parents identified as suitable for recruitment were sent a letter prior to their child's clinic appointment explaining the study rationale and what was involved. Parents were then randomly allocated (by simple randomisation) to one of two groups:

1. ‘Model group’: a 3D patient-specific model was manufactured for use during each visit;
2. ‘Control group’: no model was used during consultation.

The parents gave informed consent to take part in the study; however, due to the requirement to print the models in advance of the clinic visit, parents were randomised before they had been consented.

As data on parental knowledge is sparse, it was difficult to estimate differences between the groups and SDs in order to power the study appropriately; a power calculation was instead performed retrospectively to verify that the study was sufficiently powered for the observed results to be statistically significant.

### Model manufacturing

The 3D patient-specific models were derived from medical imaging data. For CHD, the most commonly used imaging modalities are cardiac magnetic resonance (CMR) or CT. In the present study, CMR data alone were used, to avoid having to accommodate substantial differences in spatial resolution between the two modalities. CMR studies were performed based on medical indication and were retrieved retrospectively for the 3D reconstructions. Timing between CMR and consultation was 1.7±1.6 years.

The process of model manufacturing for this specific application has been described and discussed in detail elsewhere.^18^ ^19^ Briefly, CMR data are segmented using the commercial software Mimics (Materialise, Leuven, Belgium), the thresholding being based on the pixel greyscale. Then, following operations of segmentation and region growing, the 3D model of the desired area can be obtained and, if necessary, surface irregularities can be smoothed. Having added an arbitrary wall thickness of 1–1.5 mm to ensure model robustness using another type of software (Abaqus V.6.13, Dassault Systèmes, Waltham, Massachusetts, USA), the model can finally be exported in a stereolithography (.stl) file format to the printer. The 3D printer used for the models in this study was an EOSINT P360 machine (EOS Electro Optical Systems, Krailling, Germany) based on selective laser sintering (SLS) technology.^20^ All models were produced in white nylon, chosen as a neutral colour/material.

**Figure 1:**
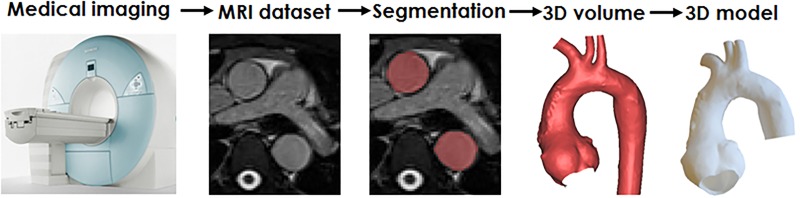
Illustrating the steps for manufacturing a three-dimensional (3D) patient-specific model, the example showing a patient with an enlarged Marfan-like aortic root.

**Figure 2:**
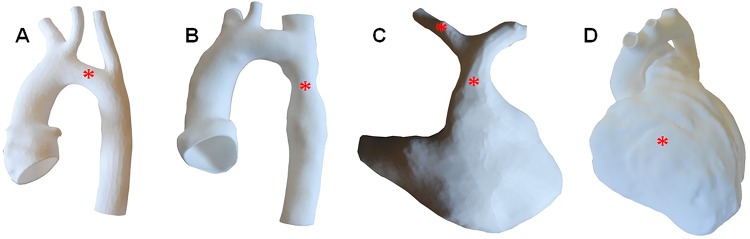
Examples of models produced for the study: (A) patient with hypoplastic transverse aortic arch; (B) patient with aortic coarctation; (C) pulmonary anatomy of a patient being assessed for percutaneous pulmonary valve intervention, showing a hypoplastic right pulmonary artery, the left pulmonary artery and the right ventricular outflow tract and (D) patient with repaired tetralogy of Fallot presenting with dilated right ventricle. In all cases, the red star(s) indicate(s) the lesion(s) being discussed. Models not to scale.

A visual summary of these steps is provided in figure 1. Examples of models of congenital defects from this study are provided in figure 2.

### Procedure

On arrival at the clinic, parents who had agreed to participate were asked to sign a consent form and to complete a brief questionnaire before their child's consultation. In cases in which both parents of a child were attending, only one was asked to participate. Parents were asked to rate their understanding of their child's heart condition on a scale from 1 (‘extremely poor’) to 10 (‘extremely clear’), and to name and identify their child's heart defect(s). Parents were able to mark information on a diagram of the heart, identify relevant words from a list of key words and provide free text answers. They were also asked to provide some basic demographic information (relationship to patient, age and highest level of education). Questionnaires took less than 5 min to complete and were anonymised.

Following the consultation, parents were asked to complete a second brief questionnaire that included the same questions about their understanding and description of their child's heart defect. There were also questions about the clarity of the explanation of their child's condition that they had been given and, if applicable, about any planned procedure or intervention (rating from 1 ‘extremely unclear’ to 10 ‘extremely clear’). They were also asked to rate any tool that the cardiologist used during the consultation, such as video, drawings, leaflets, 3D model and medical images (ie, echocardiography, CMR and X-rays). Participants were also given the opportunity to provide free text comments.

Additionally, cardiologists were asked to rate how clearly they felt the parent understood their child's anatomy and, if applicable, the procedure. If they used a 3D patient-specific model, they were also asked whether it took a long time to explain, whether it helped with communication (1–10 rating) and how well the participant engaged with the model (1–10 rating).

The duration of each consultation was timed for an objective evaluation to be compared with the clinicians’ subjective evaluation on whether the use of a 3D model impinged or not on this time.

### Sample size

Pilot data were not available to power the study. A retrospective power calculation based on clinicians’ rating, as a more objective measure than perceived parental knowledge, showed that given the observed mean difference, SDs and the number of participants included in each group, the study was sufficiently powered at 80% with p=0.05.

### Data analysis

**Figure 3:**
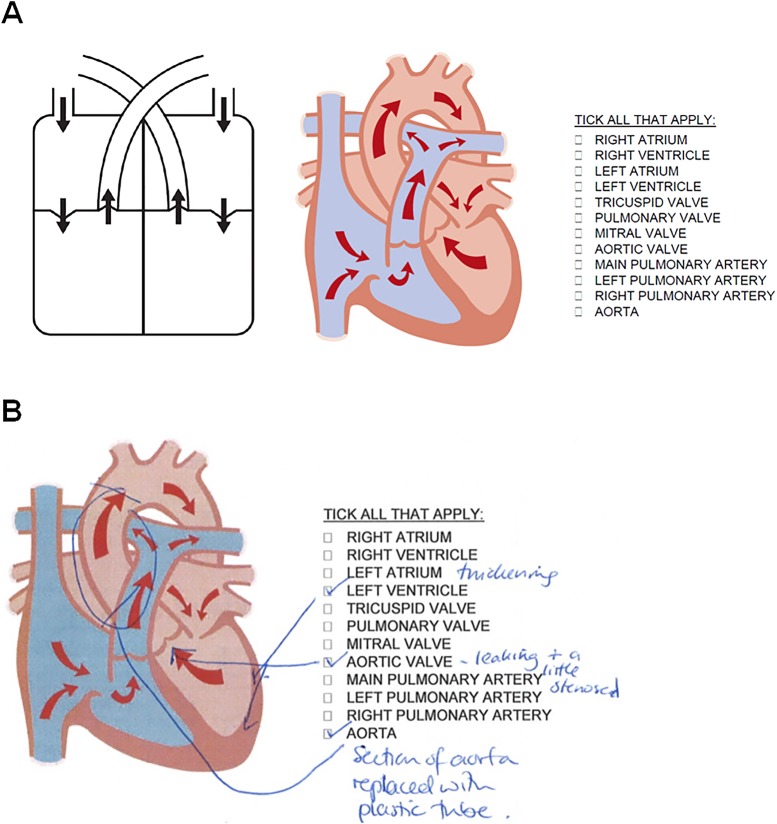
(A) Two diagrams were provided in the questionnaires administered to the parents, representing the simplified cardiac anatomy. Parents were encouraged to use the diagrams to mark the location of the defect(s) in their child's heart/vasculature. As an additional tool, a list of keywords was provided next to the diagrams. (B) Example from a completed questionnaire.

With regard to parental knowledge, three different measures were used to obtain indicators of their understanding of their child's heart condition, as follows:

- *Subjective*: as indicated by the parents themselves in the responses in the questionnaires (ie, perceived knowledge, 1–10 rating) and as indicated by cardiologists immediately after the consultation;
- *Division into classes*: two blinded researchers separately evaluated all parent questionnaires (both before and after the consultation) and based on their responses, parents were divided into four classes, considering ‘good knowledge’ as the parents being able to describe appropriately their child's condition:
  - Class I: good or very good knowledge (correct name of diagnosis, use of medical language, correct identification of the defect on the diagram);
  - Class II: adequate knowledge (description sufficient to decipher the diagnosis, at least in part, using lay language, eg, ‘narrowing’ instead of ‘stenosis’ or ‘hole in the heart’ instead of ‘atrial/ventricular septal defect’);
  - Class III: vague knowledge (some indication of the diagnosis by identifying a correct keyword, eg, ‘pulmonary valve’ for ‘tetralogy of Fallot’, however, not sufficient to describe the condition in full);
  - Class IV: poor knowledge (blank response, incorrect keywords, incorrect identification on the diagram)
- *Blind assessment from clinicians*: all the answers to previously anonymised questionnaires were typed and diagrams, if used, were scanned (figure 3B). Such recompiled questionnaires were given in a random order to two cardiologists who were not involved in the previous consultations, and who were asked whether they would be able to gather the correct diagnosis based on the information provided and how they would rate the level of knowledge in each case.

Furthermore, qualitative assessment of the feedback provided by the parents was carried out. Feedback and comments were grouped by themes.

Differences between continuous variables in the two groups were assessed with two-sample Student t test and differences between groups in terms of sex (male vs female) and education (low vs high, with high defined as university graduate + university postgraduate) were assessed with χ^2^ test. Analysis was performed in Stata (V 13.1, College Station, Texas, USA).

## Results

Of the 103 parents approached for participation, 6 refused because they were too distressed prior to their child's appointment, resulting in 45 assigned to the Model group and 53 to the control group. Demographics of the participants are reported in table 1 and the primary diagnoses of all patients included in the study are reported in table 2. In three cases, all from the model group, the patient attended clinic without the parent. In these instances, data were only collected from the clinicians.

**Table 1:** Study demographics

Variables	Model group (n=45)	Control group (n=52)	p Value
Parental age (years)	44±7	41±9	0.1
Patient age (years)	14±5	10±6	0.001
Sex (F/M)	33/9	40/12	0.8
Level of education (n)			
6th form	13	15	
After 6th form	7	7	
University graduate	9	11	
University postgraduate	4	8	
Other	9	10	
Total	42*	51*	

Note: 6th form is equivalent to 12th grade in the USA.

*One participant in the control group did not want to disclose this information, while the missing three from the total in the intervention group refer to those three cases in which the patients attended the clinic without their parents.

F, female; M, male.

**Table 2:** List of diagnoses of the cases that were randomly assigned to each of the two groups

Diagnosis	Model group (n=45)	Control group (n=52)
Aortic coarctation	7	9
Pulmonary stenosis/atresia	5	9
Fontan type circulation	8	8
Tetralogy of Fallot	8	7
Transposition of the great arteries	10	6
Aortic stenosis	1	2
Marfan syndrome	2	0
Bicuspid aortic valve	2	0
Ventricular septal defect	0	2
Atrial septal defect	0	2
ALCAPA	0	2
Total anomalous pulmonary venous drainage	1	0
Aortic interruption	0	1
Double-inlet left ventricle	1	0
Kawasaki	0	1
Williams syndrome	0	1
AV valve regurgitation	0	1
PDA with left SVC	0	1
*Total*	*45*	*52*

ALCAPA, anomalous left coronary artery from the pulmonary artery; AV, atrioventricular; PDA, patent ductus arteriosus; SVC, superior vena cava.

Results from the questionnaires are summarised in table 3. Generally, parents rated their knowledge highly, before and after the consultations. A slightly higher, but not statistically significant improvement in perceived knowledge was noted in the group who had access to the model (1.2±1.3 vs 0.8±1.5 points, p=0.2). Parents in both groups found that the explanation they were given during the visit was very clear. Overall, parents rated the 3D models as ‘very useful’, and in 73% of cases (33/45) explicitly asked whether they could keep the model, which was then gifted to them at the end of the study. This was taken as qualitative evidence that the models were well liked by parents.

**Table 3:** Summary of results

Variable	Model group (n=45)	Control group (n=52)
Parent assessment		
Self-assessed knowledge (before)	7.9±1.6	8.1±1.7
Self-assessed knowledge (after)	9.1±1.1	9.0±1.2
Clarity of explanation received	9.3±1.1	9.1±1.3
Usefulness of 3D model	9.5±0.7	–
Cardiologist assessment		
Parent knowledge (after)	7.0±1.9	8.0±1.7
Quality of interaction with model	9.1±1.4	–
Usefulness of 3D model	8.8±1.1	–

All values are derived from the questionnaires answered by the parents and by the cardiologists. Values are on a scale of 1–10 with 1 indicating the lowest score and 10 indicating the highest score.

3D, three-dimensional.

Clinicians felt the parent knowledge was adequate, although scoring it lower than the parents themselves. Clinicians rated the models as ‘very useful’, and remarked that the users interacted very well with the 3D models. The clinicians noted that they needed a long time to explain the 3D model in only 7% of cases (3/45), overall remarking that using the models does not impinge on the duration of the visit. In actuality, consultations with the 3D model lasted on average 5 min longer than those in which the model was not employed (21±10 vs 16±7 min, p=0.02).

**Figure 4:**
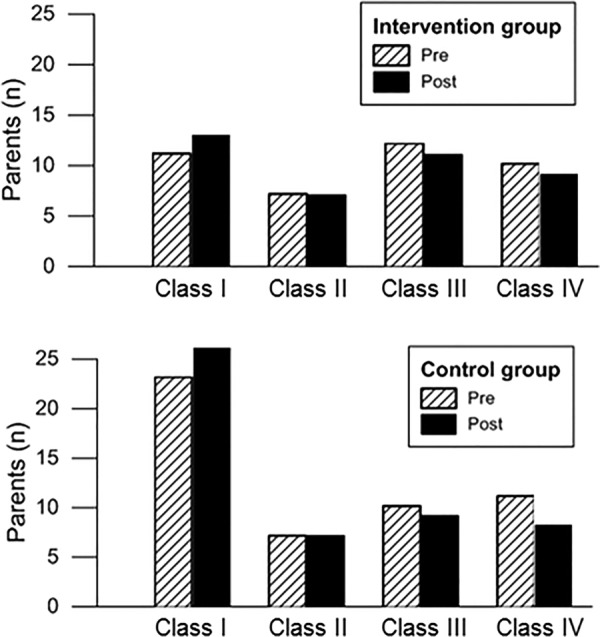
Parental knowledge was also assessed by grading their responses into classes I–IV, where I=good/very good knowledge, II=adequate knowledge, III=vague knowledge and IV=poor knowledge (criteria are detailed in Materials and Methods section). A small increase in class I was noted comparing parent responses ‘pre’ and ‘post’ the consultation, indicating a small increment in knowledge, with a similar trend observed in both groups (model group vs control group).

Parent understanding rated according to the division into classes showed only a moderate improvement after the consultations (5 more cases in class I for both groups, figure 4). Good knowledge appeared not to be significantly associated with the parental level of education, as parents with a good level of knowledge (class I) were not necessarily those with a higher degree of education (only 41% of class I parents had high education, across the two groups; p=0.2).

According to the blind assessment by two cardiologists, it was not possible to identify the primary diagnosis in 41.5% of cases; this contrasted significantly with parental perceived knowledge prior to the visits. This did not improve following the consultations, with 40.5% of the cases in which the clinicians were still not able to gather a primary diagnosis. The clinicians marked an improvement in clarity of the description provided in the questionnaire following the visit in 5 cases in the model group and 12 cases in the control group.

Box 1 **Participant feedback on three-dimensional (3D) models**

Medical images are difficult to interpret:

“*I don't understand his [my son's] condition as I've never had it explained and it's very complex*” (Study Q038, mother of 14-year-old male)

Medical images are “*useful to him [the clinician] but not to me*” (Study Q043, mother of 7-year-old male)

“*Medical terms mean nothing to a 16 year old*” (Study Q080, mother of 16-year-old male)

“*Medical images are difficult to interpret*” (Study Q082, father of 16-year-old male)

3D model is more immediate:

It was “*incredibly useful to our understanding of [his] heart issues to have the Dr use a model rather than draw a sketch, which I've found a little meaningless if I'm honest. To see a model, 3D, is realistic and much easier to understand. To have a 3D model made of [his] pulmonary arteries is unique, amazing (to us) and something we have never seen before. Echo scans for example are important for the doctors but meaningless visually to us. This 3D model is the opposite of that and makes it all so much easier for the non-expert to understand*” (Study Q086, mother of 14-year-old male)

The “*3D model really helped my son to understand*” (Study Q103, mother of 17-year-old male)

There could be some initial shock when looking at a realistic model:

“*Seeing the model of my son's heart (from the MRI) was quite a shock. Looking at images of what his condition could look like was somewhat better, more impersonal, although once I was used to the idea that the model I was looking at was what my son's heart actually looked like, it was a very useful tool for the doctor to illustrate exactly what was wrong with his heart*” (Study Q096, mother of 16-year-old male)

Also, from the patients’ side, in one case it was reported that the patient was “*anxious about looking at her [own] heart*” (Noted by the clinician, Study Q040, 16-year-old female)

The model can stimulate curiosity:

In the case of a patient with a complex repair (left-sided total cavopulmonary connection) who came alone to the appointment, it was noted that “*he was not interested to know about his condition until he was shown the model*” (noted by the clinician, Study Q065, 15-year-old male)

The model would be more useful when explaining for the first time:

“*As we are near the end of our treatment [this is] not as important as at the diagnosis stage*” (Study Q055, father of 17-year-old male)

The model would be more useful when compared with a reference (normal anatomy):

“*It would be good to have an anatomical model of a normal heart and then shown the areas that have the problem*” (Study Q082, father of 16-year-old male)

Parent feedback is reported in box 1. The main points that emerged are:

1. Medical images are difficult to interpret and do not aid parental understanding;
2. 3D models are more immediate to understand than echocardiograms or sketches;
3. The models can be shocking at first, when the parents realise it is the actual anatomy of their child and not a generic model;
4. The model can stimulate curiosity (ie, better engagement than other media), especially in youngsters; and
5. 3D models might be especially helpful at the time of the initial diagnosis, combined with a reference model (ie, normal cardiac anatomy).

## Discussion

To the best of our knowledge, this is the first study that has attempted to quantify the advocated, but never systematically demonstrated benefit of 3D patient-specific models in the realm of doctor–patient communication. Specifically, a group of parents of children with CHD was targeted as a particularly interesting and challenging scenario in which to assess the potential of the 3D models, manufactured with the technique known as rapid prototyping (ie, ‘3D printing’). The rationale of the study was that, given the complexity of repaired CHD (in terms of anatomy, future/additionally required treatment and non-generalisability of the conditions), a real replica of the area of interest would be helpful for the parents to better understand, manipulate, and ultimately visualise:

- Where the anatomical structures of interest are located and are positioned with respect to each other;
- A specific area that the cardiologist is describing (eg, appreciating the severity of a narrowing in a vessel);
- What the repair has been or what it will entail (eg, the area where a stent was inserted or might be inserted in the future).

Parents responded enthusiastically to the use of 3D models. This transpired both from the questionnaires that they completed after their consultations—scoring the 3D models as very helpful tools—and from the feedback they were encouraged to provide. In particular, parent feedback highlighted that 3D models are perceived as more immediate and user-friendly than medical images (eg, echocardiograms), whose importance the parents recognised (for the clinician) but which they themselves found difficult to comprehend. Furthermore, in three quarters of cases, parents asked to keep the model, which might be taken as a sign of their appreciation of and interest in the models.

Cardiologists also rated the models as very useful for engaging parents in discussion of their child's condition and noted generally that parents interacted well with the 3D models.

As a communication tool, models may lead to a more detailed explanation being provided, and this was borne out by the finding that consultations involving the use of a model lasted, on average, 5 min longer. This additional time is unlikely to impinge significantly from a clinical point of view on the overall duration of the visits. Indeed, clinicians did not feel that consultations involving a model lasted longer than usual. However, although the longer consultation may mean that more detailed information was transmitted as a result of the models, it cannot be claimed that 3D models reduce consultation times by making communication more effective.

Despite parental appreciation for the models, there was no objective evidence that parents' understanding of their child's heart condition was improved. Their perceived knowledge as well as a basic quantification of their understanding from the questionnaires did not indicate a marked increase in the group in which 3D models were used. Knowledge is, however, very difficult to quantify. While the questionnaires provide some indication of overall parental understanding of the anatomy and the condition (ie, correct name of diagnosis or correct use of diagrams), a deeper appreciation of the complications and lifestyle implications does not emerge from this kind of assessment. However, their feedback is important and highlights:

- Their difficulty in relating to medical images (seen as a tool more for the expert than for the non-expert) and sketches (which may be intuitive in some conditions, eg, septal defects, but less in others, eg, complex circulations such as Fontan or repaired transposition of the great arteries);
- Their concern with facing the real anatomy of their child (ie, an element of initial shock), which is then balanced by the perceived improved understanding;
- The need for a reference model to better appreciate what is abnormal or what has been modified by surgery/interventions, a point that will be included in our future work.

An interesting dichotomy also emerged from this study, which, while focused on assessing the 3D models, also looked at the clinician–parent interaction, overall. In fact, while parents felt that the received explanations were always extremely clear, in 40% of cases, blinded clinicians were not able to identify even the primary diagnosis based on information provided by the parents after the consultation! Albeit a strikingly high proportion, this is interestingly in keeping with previous observations in the literature. A 2004 questionnaire survey focused on parental understanding of CHD reported that, out of 156 parents, only 59% could correctly name their child's CHD, and only 29% could correctly indicate the lesion on a diagram.^21^

Models were all printed in white nylon. This was considered to be a neutral option, especially for a first evaluation of this kind of communication device. However, it may be interesting to explore the use of colour in model manufacturing. Coloured models may result in more intuitive representations, for example, red and blue rendering for veins and arteries, respectively, which is often used even in non-specialist textbooks and websites.

This study does not include a detailed cost-effectiveness analysis, as it focused on assessing the feasibility of using models in clinical practice, and the acceptability of the tool from the parent and clinician perspectives. The models printed in this study each cost approximately £50 to make. It should be noted that price varies depending on the size of the model, reflecting the amount of material being used; this study comprised a wide range of sizes (eg, from infants with hypoplastic left heart syndrome to teenagers with tetralogy of Fallot) and thus a broad price range. Nevertheless, the average price appears reasonable and it might be desirable to perform a cost analysis in the future to investigate this aspect further. It is also sensible to speculate that, with the very recent technical advances in 3D printing, models might be cheaper in the near future, although their quality for this specific medical application would need to be verified. From a practical perspective, it is feasible to produce these patient-specific models in a timely manner: image reconstructions take approximately 0.5–3 h, depending on the image quality and the complexity of the anatomy being reconstructed, while printing time is of the order of 24 h.

This study has focused on a relatively small population composed exclusively of parents of children with CHD. As a next step, it would be interesting and important to involve the patients themselves, perhaps initially targeting the teenage population. The possibility that keeping the model as a reminder of the anatomy might lead to long-term improvements in the parents’ and patients’ understanding of the condition should also be tested. This may be particularly important for future medical consultations where the parents or patients may need to communicate the nature of the diagnosis to healthcare professionals who are not aware of it. Indeed, the literature has reported that a large number of parents of children with CHD lack knowledge regarding lifelong congenital cardiac care but demonstrate a desire to learn.^22^ Personalised models could facilitate this learning process, with a potential long-term impact on lifestyle adjustments that would be important to observe and quantify in future studies.

From a psychological standpoint, other factors (eg, deflecting anxiety) could also be investigated in future studies.

While it would be interesting to assess the usefulness of 3D models for specific diagnoses or interventions (eg, pulmonary valve replacement), the number of cases in this study is too small to allow for subgroup analysis.

## Conclusion

Parents of children with CHD and cardiologists both appreciated the use of 3D patient-specific models during routine/follow-up consultations. “I'm still looking at the heart [model] in absolute amazement and am guarding it like a Doberman! [...] It really does help knowing and understanding what is planned” (*email communication from one of the parents of a patient*). These models are useful for enhancing engagement with parents and, importantly, for improving communication between cardiologists and parents. In turn, this may also have a positive impact on parents’ and patients’ psychological adjustment to living with CHD.
